# Toward Consensus Epitopes B and T of Tropomyosin Involved in Cross-Reactivity across Diverse Allergens: An In Silico Study

**DOI:** 10.3390/biomedicines12040884

**Published:** 2024-04-17

**Authors:** Dalgys Martínez, Luis Fang, Catherine Meza-Torres, Gloria Garavito, Guillermo López-Lluch, Eduardo Egea

**Affiliations:** 1 Department of Medicine, Health Sciences Division, Universidad del Norte, Barranquilla 081007, Colombia; inmunolab01@uninorte.edu.co (D.M.); lfang@uninorte.edu.co (L.F.); inmunolab02@uninorte.edu.co (C.M.-T.); ggaravit@uninorte.edu.co (G.G.); 2 Institute for Immunological Research, University of Cartagena, Cartagena 130014, Colombia; 3 Department of Physiology, Anatomy, and Cellular Biology, Andalusian Centre for Development Biology (CABD-CSIC-JA), Pablo de Olavide University, 41013 Seville, Spain; glopllu@upo.es; 4 Health Sciences Division, Universidad Simón Bolívar, Barranquilla 080002, Colombia

**Keywords:** allergen, IgE epitope, B-cell epitope, T-cell epitope, cross-reactivity, tropomyosin

## Abstract

Tropomyosin (TM) is a pan-allergen with cross-reactivity to arthropods, insects, and nematodes in tropical regions. While IgE epitopes of TM contribute to sensitization, T-cell (MHC-II) epitopes polarize the Th2 immune response. This study aimed to identify linear B and T consensus epitopes among house dust mites, cockroaches, *Ascaris lumbricoides*, shrimp, and mosquitoes, exploring the molecular basis of cross-reactivity in allergic diseases. Amino acid sequences of Der p 10, Der f 10, Blo t 10, Lit v 1, Pen a 1, Pen m 1, rAsc l 3, Per a 7, Bla g 7, and Aed a 10 were collected from Allergen Nomenclature and UniProt. B epitopes were predicted using AlgPred 2.0 and BepiPred 3.0. T epitopes were predicted with NetMHCIIpan 4.1 against 10 HLA-II alleles. Consensus epitopes were obtained through analysis and Epitope Cluster Analysis in the Immune Epitope Database. We found 7 B-cell epitopes and 28 linear T-cell epitopes binding to MHC II. A unique peptide (residues 160–174) exhibited overlap between linear B-cell and T-cell epitopes, highly conserved across tropomyosin sequences. These findings shed light on IgE cross-reactivity among the tested species. The described immuno-informatics pipeline and epitopes can inform in vitro research and guide synthetic multi-epitope proteins’ design for potential allergology immunotherapies. Further in silico studies are warranted to confirm epitope accuracy and guide future experimental protocols.

## 1. Introduction

Allergic diseases have emerged as a public health problem, with an increased risk of developing diseases attributed to a combination of genetic (atopy) and environmental factors [1]. House dust mites (HDMs), shellfish, cockroaches, insects, and nematode allergens such as *Ascaris lumbricoides* are common allergens causing allergic diseases in tropical areas [2,3]. Tropomyosin (TM) is considered a clinically significant allergen because it is classified as a pan-allergen with a high degree of cross-reactivity between several allergens from different species [4,5]. 

Tropomyosin is a heat-stable protein that adopts an alpha-helical coiled-coil dimeric structure and has a molecular weight ranging from 34 to 38 kDa, depending on the species [4,6]. It is an essential component of allergic reactions to crustaceans (Lit v 1, Pen m 1, and Pen a 1) [7,8], invertebrates such as HDM (Der p 10, Der f 10, and Blot 10) [9], cockroach *Periplaneta americana* (Per a 7) [10], mosquito *Aedes aegypti* (Aed a 10) [11], and nematodes such as *A. lumbricoides* (rAsc l 3) [12,13]. This allergen can induce sensitization via various routes, including ingestion (seafood) and inhalation (HDM and cockroaches) [1].

The sensitization frequency to this allergen varies widely across different geographical areas, ranging from 2% in Singapore to 29% in European countries [14]. In Colombia, more than 35% of HDM-allergic individuals are sensitized to mite TM [12] and in Martinique, France, the frequency of positive IgE reactivity to TM of HDM species was reported to be 82.35%, for *A. aegypti* 64.7%, *P. americana* 29.4%, and *L. vannamei* 23.5% [15]. In addition, Ascaris infection has been found to increase IgE reactivity to specific mite allergens in patients with asthma. Tropomyosin has been recognized as a critical player in the cross-reactivity between HDM, shellfish, cockroaches, mosquitoes, and Ascaris [5,9,13,15,16,17,18]. 

Cross-reactivity (CR) is an immunological phenomenon that occurs when an antibody directed against a specific antigen recognizes shared epitopes or the conformational similarity of these epitopes in the three-dimensional structure of other allergens [19,20]. CR is an immunological phenomenon induced by humoral (IgE antibodies) and T-cell responses. IgE cross-reactivity arises when antibodies bind to conformational epitopes similar to the allergen that causes sensitization or usually with linear epitopes with high amino acid identity (>70%) among the recognized allergens. In comparison, cross-reactivity between proteins with less than 50% sequence identity is rare. It has been reported that allergens are closely linked or are the same species [19,21]. T-cell cross-reactivity occurs when linear peptides from different allergens share key residues bound to MHC-II [22], thereby enabling recognition by the same T cell. Effector T cells produce Th2 cytokines that trigger the B cell to switch to IgE and produce allergen-specific IgE [23]. Cross-reactivity has significant clinical and epidemiological implications and can expand the repertoire of allergens recognized by patients with allergies that affect allergen-specific immunotherapy [20]. 

Although the cross-reactivity of tropomyosin among different species has been extensively investigated, at the level of B-cell epitopes (IgE-binding epitopes), as well as the protein sequence, it is widely recognized that non-IgE-mediated allergies can be triggered by T cells. Therefore, identifying pairs of peptides that can bind to class II MHC molecules and simultaneously interact with IgE could have significant implications for allergic symptoms. Moreover, this could impact treatment strategies, especially in allergy immunotherapy, as a peptide with the ability to interact with both components of the immune system could be exploited to modulate immune responses and develop more specific therapeutic approaches. The specific purpose of this in silico study was to identify novel B-cell and linear T-cell consensus epitopes between different tropomyosins from shrimp, HDM, *A. lumbricoides*, and cockroaches that play a role in the sensitization of atopic patients and to explain the cross-reactivity between different species.

## 2. Materials and Methods

Amino acids from 10 tropomyosin sequences from HDM (*Dermatophagoides pteronyssinus* (Der p 10), *Dermatophagoides farine* (Der f 10), and *Blomia tropicalis* (Blo t 10)), shrimps (*Litopenaeus vannamei* (Lit v 1), *Penaeus aztecus* (Pen a 1), and *Penaeus monodon* (Pen m 1)), *Ascaris lumbricoides* (rAsc l 3), cockroaches (*Periplaneta americana* (Per a 7) and *Blattella germanica* (Bla g 7)), and mosquito *Aedes aegypti* (Aed a 10) were collected from Allergen Nomenclature website (http://allergen.org, accession date: 21 July 2023) of the World Health Organization and International Union of Immunological Societies (WHO/IUIS), and the Universal Protein Resource—UniProt (https://www.uniprot.org, accession date: 21 July 2023) according to the accession number. We considered only complete sequences. The 3D models of tropomyosins described above were taken from the AlphaFold database (https://alphafold.ebi.ac.uk, accession date: 21 July 2023).

### 2.1. Phylogenetic and Multi-Alignment Analyses

Homology between tropomyosins was estimated using multiple sequence alignment in CLUSTAL-Ω (EMBL-EBI) (https://www.ebi.ac.uk/Tools/msa/clustalo/, accession date: 21 July 2023); only identical or functionally similar amino acid residues were considered for calculating the conservation scores [24]. Subsequently, sequence identity and similarity (SIAS) were calculated using the default parameters (http://imed.med.ucm.es/Tools/sias.html, accession date: 21 July 2023). To calculate the normalized similarity score, the BLOSUM62 matrix was chosen. A phylogenetic tree was constructed using Molecular Evolutionary Genetic Analysis (MEGA 11) with the neighbor-joining method and 1000 replicates to measure reliability and robustness under the premise of an evolutionary minimum [25].

### 2.2. Conservation Analysis of Tropomyosins

Conservation analysis of amino acid residues for each tropomyosin was performed using the ConSurf web server (https://consurf.tau.ac.il/consurf_index.php, accession date: 21 July 2023) [26], which computes position-specific conservation scores using an empirical Bayesian algorithm from the multiple sequence alignment matrix obtained using CLUSTAL-Ω. Conservation scores were divided into 9 categories, ranging from the most variable positions (grade 1, colored turquoise) to the most conserved positions (grade 9, colored maroon). The number of species-specific amino acid residue matches was plotted on a heat map using Heatmapper, which employs Euclidean distance and average-linkage clustering methods [27]. 

### 2.3. Prediction of Linear B-Cell Epitopes and IgE Epitopes

All tropomyosin amino acid sequences were analyzed using two B-epitope prediction methods. AlgPred 2.0 (http://www.imtech.res.in/raghava/algpred/submission.html, accession date: 28 August 2023) was used to map and predict amino acid sequences that are potentially allergenic (IgE epitopes) based on the similarity of known IgE epitopes by mapping IgE epitopes, PID (Percent Identity Difference), and ARP (Allergen-Representative Peptides) (http://www.imtech.res.in/raghava/algpred/submission.html, accession date: 28 August 2023) algorithms [28]. BepiPred 3.0 server (https://services.healthtech.dtu.dk/services/BepiPred-3.0/, accession date: 28 August 2023) validated the IgE epitopes identified with AlgPred 2.0 and predicted B epitopes using neural-network-trained protein language models; for all predictions, a cutoff of 0.15 and higher confidence was set up [29]. Both methods predict B epitopes based on the physicochemical properties of amino acids. B epitopes were defined by combining data from these two prediction methods. Our selection of peptide regions for analysis focused on those predicted as IgE and linear B-cell epitopes. This choice was guided by the filamentary structure predominantly comprising alpha helices in tropomyosin, leading to a correspondence between conformational and linear epitope regions. It is well-documented that the IgE-binding epitopes within this protein primarily manifest as linear epitopes. Consequently, we decided to consider linear sequences exclusively. B epitope consensus between species was obtained by epitopes conservation analysis tool available in Immune Epitope Database—IEDB (http://tools.iedb.org/conservancy/, accession date: 28 August 2023). The consensus was considered if the sequence presented a 100% identity percentage in at least two species [30]. 

### 2.4. Prediction of T-Cell Epitope/MHC-II Binding

T-cell cross-reactivity has been less studied than IgE cross-reactivity and depends on MHC ligands and peptide-TCR recognition; therefore, the prediction of peptide binding to MHC is a powerful tool to predict the possible specificity of a T-cell immune response. We employed the MHC-II binding prediction tool available in IEDB (http://tools.iedb.org/mhcii/, accession date: 28 August 2023) to predict peptide regions binding to MHC-II using NetMHCIIpan 4.1 EL (Eluted Ligand mass spectrometry); NetMHCIIpan 4.1 was released in 2023 and use Artificial Neural Networks (ANNs) to predict peptide binding to any MHC II molecule of known sequence [31]. For this prediction, fragments of 15 amino acids long and a panel of 10 HLA-II alleles that have been associated with allergic diseases in different communities were taken into account: HLA-DRB1*03:01, *04:01, *04:04, *04:05, *11:01, *11:04, *14:01, *15:01, *16:02, HLA-DQA1*05:01/DQB1*02:01, and DQA1*03:01/DQB1*03:02 [32,33,34,35,36]; the epitopes candidates were classified as either a strong binder (<1 percentile rank), weak binder (≤percentile rank 5), or no binder (≥5 percentile rank). The CD4 T-cell immunogenicity prediction tool available in IEDB-predicted immunogenic peptides can be recognized by TCR (T-cell receptor) [37]. T-cell epitopes were defined by combining the data from these two prediction methods. We chose peptide regions predicted to be ligands for MHC-II, which the TCR of CD4 T cells can recognize [38].

### 2.5. B-Cell and T-Cell Epitope Consensus

IEDB’s Epitope Cluster Analysis tool was employed to group the predicted epitopes (http://tools.iedb.org/cluster/, accession date: 28 August 2023). A cluster is a collection of sequences sharing a sequence similarity exceeding the specified minimum sequence identity threshold. The identity set percentage signifies that any member within the cluster must be at least the designated percentage identical to at least one other cluster member. In this study, a cluster was defined as an assembly of sequences demonstrating a sequence similarity > 70%, and breaking the connected clusters to obtain a precise consensus sequence represents the default setting for cluster analysis (http://tools.iedb.org/cluster/help/, accession date: 28 August 2023) [30]. Finally, the spatial distributions of B- and linear T-cell epitopes identified by computational tools were mapped onto 3D models of tropomyosin targets using PyMOL software (The PyMOL Molecular Graphics System, Version 2.3.2 Schrödinger, LLC, New York, NY, USA) using the cluster-break method to obtain clear representative consensus sequences with at least 70% homology [39].

## 3. Results

**Figure 1:**
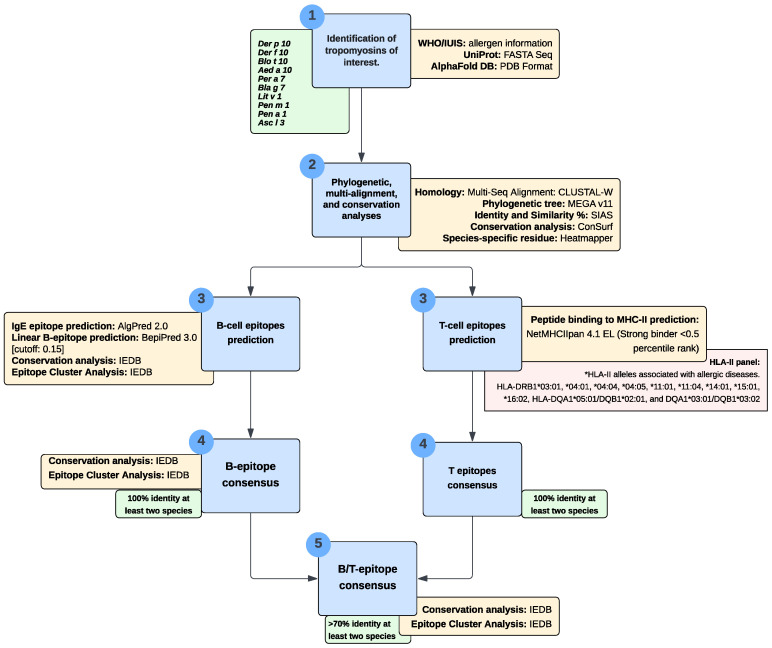
The flow chart of epitope prediction analysis applied to predict B-cell and T-cell epitopes.

We have meticulously designed a robust immune-informatics pipeline, as illustrated in Figure 1, to predict consensus epitopes for both B-cell and T-cell responses against tropomyosin. This process involved meticulously selecting and applying diverse bioinformatics tools and databases. The subsequent subsections meticulously outline detailed outcomes from each stage of this comprehensive pipeline (Figure 1).

### 3.1. Analysis of the Amino Acid Sequence of Tropomyosin from Different Species

The amino acid sequences and structural 3D models of tropomyosin from different species, such as HDM, shrimp, nematode, cockroach, and mosquito, were obtained from the UniProt protein database and AlphaFold, respectively (Table 1). 

**Figure 2:**
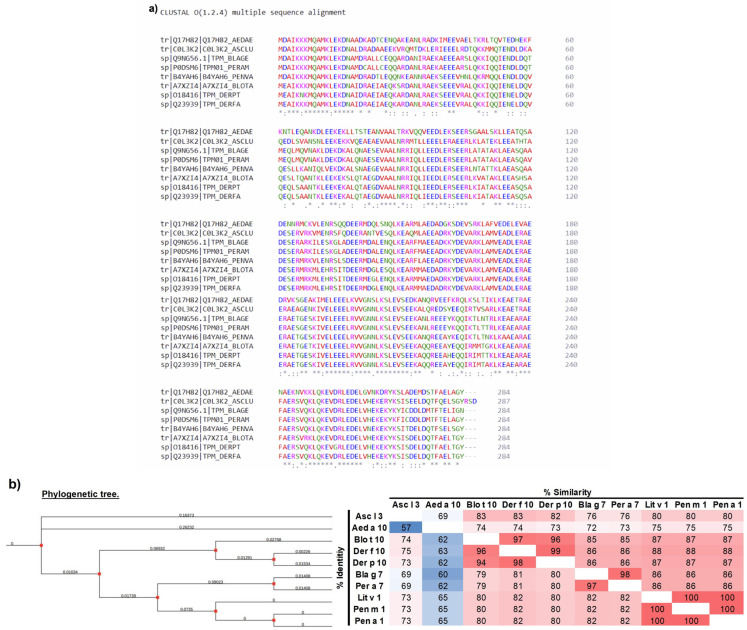
Comparison of TM’s amino acid sequence identities (%) from different species. (**a**) Multiple sequence alignment between tropomyosins of HDM, cockroaches, crustaceans, helminths, and mosquito species; identical [*], conserved [:], and semiconserved [.]. (**b**) Sequence identities and similarities were calculated with Sequence Identities and Similarities (SIAS). For the similarity calculation, the default parameters offered by SIAS were chosen. The color scale (red to white to blue) represents the percent sequence identity from greatest to least. Molecular phylogenetic tree based on published amino acid sequences of tropomyosin from the edible crustacean, HDM, cockroach species, *Aedes aegypti*, and nematodes *A. lumbricoides.* The phylogenetic tree inferred from 1000 replicates taken to represent the evolutionary history of the taxa was analyzed and constructed using the evolutionary minimum method in MEGA11. *Dermatophagoides pteronyssinus* (Der p 10); *Dermatophagoides farinae* (Der f 10); *Blomia tropicalis* (Blo t 10); *Ascaris lumbricoides* (Asc l 3); *Blattella germanica* (Bla g 7); *Periplaneta americana* (Per a 7); *Aedes aegypti* (Aed a 10); *Litopenaeus vannamei* (Lit v 1); *Penaeus monodon* (Pen m 1); *Penaeus aztecus* (Pen a 1).

Multiple alignments with Clustal-Ω and SIAS analysis showed 100% identity between the three shrimp tropomyosin sequences (Lit v 1, Pen a 1, and Pen m 1). Meanwhile, for HDM (Der f 10, Blo t 10, and Der p 10) and cockroach species (Per a 7 and Bla g 7), the identity percentages ranged between 85% and 99%. A notable degree of conservation and homology became evident when comparing various species, with identity percentages ranging from 69% to 88% across shrimp, HDM, cockroaches, and *Ascaris lumbricoides.* Previous studies have reported similar identity percentages among these species, validating the technique employed in this study [5,10,17]. However, when the *Aedes aegypti* (Aed a 10) sequence was included in the analysis, it reduced identity, ranging from 60% to 69%, compared to other tropomyosin sequences (Figure 2a,b).

Phylogenetic analysis was inferred using the neighbor-joining method in MEGA 11 and demonstrated five nodes of relationships among tropomyosin from ten species. One node corresponds to tropomyosin of the three shrimp species, a second node corresponds to the two cockroach species, and the third node corresponds to tropomyosin from three house dust mite species. These three nodes have a common ancestor, which coincides with the high degree of conservation and homology observed in multi-alignment analysis. However, the tropomyosin sequences of the nematode (*Asc l 3*) and mosquito (*Aed a 10*) appeared to be more distantly related to those of other tropomyosins (Figure 2b). These findings are consistent with those of previous studies [40].

**Figure 3:**
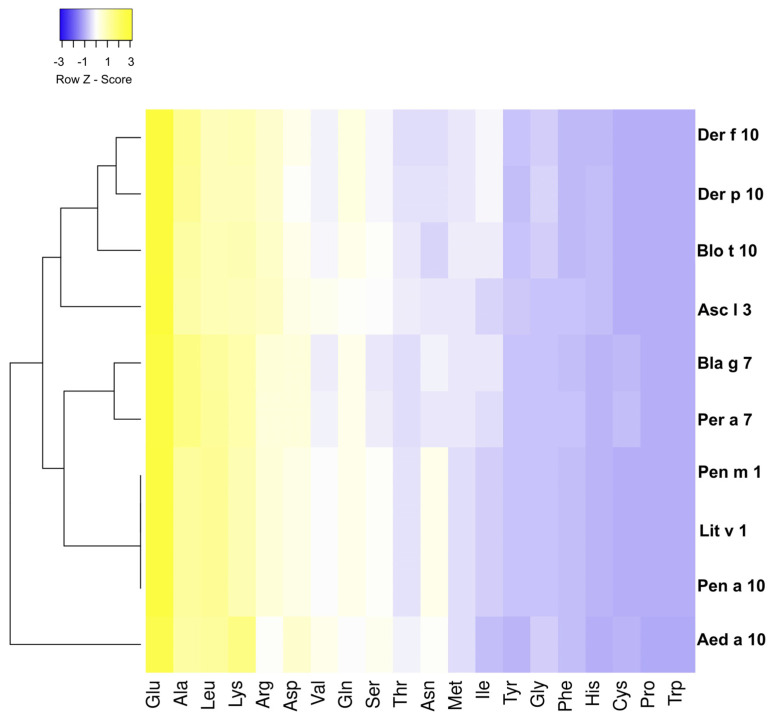
Heatmap representing the number of matches in specific residues of tropomyosin amino acid composition in different species. The Heatmap was generated using Heatmapper and clustered using the Euclidean distance metric method. The color grading, as indicated in the top left, represents the number of amino acid matches found in the specific residues of tropomyosin. Yellow indicates matches, and blue indicates maximum mismatches.

**Figure 4:**
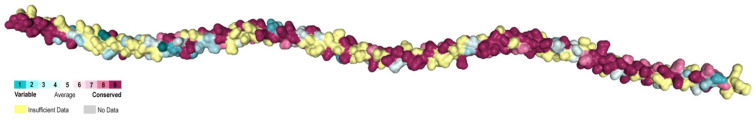
Conservation analysis of individual amino acids in tropomyosin (TM) was determined using the Consurf server. The conservation grades were subsequently superimposed onto the query sequence and structure, employing the ConSurf color-coding scheme, where the range from turquoise through maroon signifies positions from variable (grade 1) to conserved (grade 9). The conservation coloring scale is shown below.

Matching and clustering analysis of each amino acid residue of these tropomyosins revealed high concentrations of glutamic acid (Glu), alanine (Ala), leucine (Leu), lysine (Lys), and arginine (Arg), which are characteristic features of this protein family. These findings were plotted using a heat map (Figure 3). In addition, the degree of evolutionary conservation at the individual amino acid sites of each tropomyosin and its homologs was evaluated. This conservation grade, identified using ConSurf, is mapped to the query 3D structure using colors ranging from the most variable residue (grade 1, colored with turquoise) to the most conserved positions (grade 9, colored maroon). A total of 212 of 284 (74.6%) amino acid residues had conservation grades > 5, 114 (40%) had entire grade 9, and most of the conserved amino acids were located between residues 1 and 26, 155 and 217, and 233 and 259 (Figure 4). 

### 3.2. Highly Conserved B Epitopes in Invertebrate Species

Most allergens exhibit epitopes recognized explicitly by IgE antibodies, critical in diagnosing allergic reactions. This study used AlgPRED 2.0 and BepiPred 3.0 to analyze all tropomyosin sequences from various species (Supplementary Materials). We initially found 39 peptide candidates that fulfilled the selected criteria in both chosen methods. Subsequently, cluster analysis by IEDB was performed, and seven potential B-cell epitopes that are conserved sequences between species were identified (Table 2). The percentage of identity found of the seven peptides within the tropomyosin sequences of the ten allergens was between 57% and 100%. BE-02a peptide had 100% identity with tropomyosin sequence in the crustaceans (Lit v 1, Pen m 1, Pen a 1). Moreover, although the BE-02a peptide differs from BE-02b in one amino acid residue (I), it shares 100% identity with the tropomyosin sequences from HDM (Der f 10, Der p 10, and Blo t 10). The BE-05 peptide was conserved and shared by crustaceans, HDM, and Ascaris (100% identity). Furthermore, this peptide showed a 67% and 93% match between tropomyosin from *A. aegypti* and cockroaches, respectively. On the other hand, BE-07 peptide is well conserved among HDM, crustaceans, cockroaches, helminths, and mosquitoes. These conserved B epitopes could explain IgE cross-reactivity among the species studied.

On the other hand, some peptides exhibited group-specific conservation; for example, the BE-01 peptide was conserved exclusively within crustaceans, whereas BE-03 was specific to *A. aegypti*. Both peptides showed 100% sequence identity with these species (Table 2).

### 3.3. In Silico Prediction of CD4 T-Cell Epitopes and Their MHC-Binding HLA Alleles

Using IEDB’s Epitope Cluster Analysis tool, we constructed the consensus of overlapping T-cell epitope sequences, resulting in 28 potential T-cell peptides grouped into 15 clusters, Table 3. Each of Clusters 1, 11, and 14 has four epitopes. While Clusters 4, 8, and 9 each have two to three epitopes. Clusters 2, 3, 5, 6, 7, 10, 12, 13, and 15 contained only one epitope each. These T-cell peptides ranged from 16 to 25 residues in length, referred to as T-peptide 1 (TP1) to T-peptide 28 (TP28), and were predicted to have strong binding (the lowest percentile rank ≤ 0.25 higher affinity). 

Interestingly, linear T epitopes were identified as being conserved in 28.5% (8/28) of mite and crustacean species and 25% (7/28) of cockroach and Ascaris species. In contrast, only 10.7% (3/28) of the peptides were conserved in Aedes.

By identifying T-peptides that are conserved across all ten species, exhibiting a 100% sequence identity, we found several promising linear T-epitopes within the analyzed tropomyosins. Peptide TE-17 (165VARKLAMVEADLERAE180) exhibited the highest consensus, boasting a perfect 100% match with 9 of the 10 tropomyosin sequences investigated. Moreover, peptides TE-15 (158ADRKKYDEVARKLAMVE173), TE-16 (158ADKKYDEVARKLAMVE173), TE-19 (200VGNNLKSLEVSEEKA215) TE-20 (200VGNNLKSLEVSEEKAQ215), and TE-21 (VGNNLKSLEVSEEKAN) represented the second-best candidates in terms of consensus between species, showing percentage of identity with ranges between 94% and 100% in 9 of the 10 species analyzed. These peptides mentioned above showed percentages of identity between 69% and 81% with the *Aedes aegypti* tropomyosin sequence. 

Furthermore, it is noteworthy that a single amino acid differentiation between peptides TE-20 and TE-21 and TE-15 and TE-16 substantially impacted the identity percentage in relation to their respective species. 

Moreover, peptide 22 (REDSYEEQIRTVSAR) exhibited the lowest level of conservation when compared to mite (46.6%), crustacean (40–50%), cockroach (47%), and mosquito (27% identity) species, as evidenced by its percentage identity. However, this peptide demonstrated a complete match in identity with Ascaris tropomyosin, implying its specific preservation within this particular species. The differences in the nonconservative substitutions could lead to species-specific epitopes.

The peptides detailed earlier exhibit binding affinity toward six distinct alleles: HLA-DRB11:01, HLA-DQA103:01, DQB103:02, DQA103:01, DQB103:02, and DRB104:05. Notably, peptide 17 exhibited remarkable promiscuity in its binding affinity, specifically for the HLA-DQA103:01 and DQB103:02 alleles. Similarly, peptides 20 and 21 displayed promiscuous binding across the mentioned alleles, encompassing HLA-DRB1*04:05.

### 3.4. B/T Epitopes Consensus

On the other hand, when conducting consensus analyses between B and T using the IEDB program, 18 groups were identified (Table 4). There was a significant overlap between predicted B-cell epitopes and T-cell epitopes. Among these overlapping epitopes, only Groups 1, 2, 5, and 6 exhibited shared sequences between B- and T-cell epitopes. However, it is worth noting that only Group 5, which comprises peptide B05 (160RKYDEVARKLAMVEA174) and peptides TE-15 (ADRKYDEVARKLAMVE) and TE-16 (ADKKYDEVARKLAMVE), displayed consensus across mite, crustacean, cockroaches, and Ascaris species with a 100% identity percentage. Nevertheless, while this peptide did not reach 100% identity with the Aedes sequence, it exhibited an identity of over 60% in this species.

**Figure 5:**
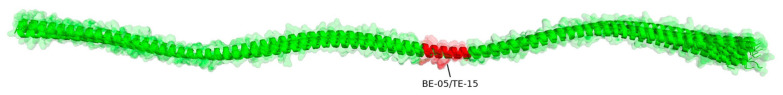
B-cell and T-cell consensus epitopes superimposition on the surface of tropomyosin allergen structure. Regions of final single B-cell and T-cell consensus epitope sequences are shown in red. Images rendered using PyMOL.

Finally, we have identified sequences BE-05 and TE-15/TE-16, which exhibit dual characteristics as both B-cell and T-cell epitopes. Notably, this sequence demonstrates consensus conservation across various species and high binding affinity for the HLA-DRB1*1101 allele. This discovery implies that the identified consensus epitope, with its dual functionality, has the potential to initiate immune responses involving both antibodies and cell-mediated mechanisms. B, T, and B/T epitopes are mapped and displayed onto the three-dimensional model of the tropomyosin of each species (HDM, shrimp, cockroach, Asc l 3, and Aed a 10) (Figure 5) of allergenic epitopes was performed using PyMOL software. 

## 4. Discussion

Elucidation of the B- and T-cell epitopes of allergens helps to understand the structure–function relationship and may predict the basis of cross-reactivity. Cross-reactive epitopes can help reduce the number of allergens without compromising the efficacy of therapy [19,20].

Tropomyosin, an extensively conserved protein found across diverse organisms, plays a pivotal role in allergy and cross-reactivity [6,15]. Its significance is underscored by its capacity to elicit allergic responses in sensitized individuals, particularly those with allergies to crustaceans (such as shrimp and lobster), arthropods (including house dust mites, mosquitoes, and cockroaches), and other related allergens. Given the structural similarities among tropomyosins sourced from different allergenic origins, individuals allergic to one specific allergen may exhibit cross-reactivity with other allergens that share analogous epitope regions [5,6,15,17]. This phenomenon of cross-reactivity carries substantial implications for precise allergy diagnosis and the development of targeted immunotherapy strategies, underscoring the critical importance of investigating and comprehending tropomyosin within the context of allergy and clinical immunology.

Immunoinformatics has emerged as a powerful tool to predict epitopes in immunogenic proteins using computational tools and biological databases. This study employed bioinformatics tools to predict B-cell epitopes, helper T-lymphocyte epitopes, and MHC class II interactions associated with allergies. We used two algorithms (AlgPred 2.0 and BepiPred 3.0 server) to predict the B-cell epitopes, and to predict peptide regions binding to MHC-II, we used NetMHCIIpan 4.1 EL of IEDB. In this study, we identified linear T-cell and B-cell epitopes that overlap between tropomyosin sequences of mites, cockroaches, shrimps, helminths, and mosquitoes, using bioinformatics methods, as well as T-cell and B-cell epitopes that show remarkable conservation between species. 

The results of the phylogenetic and SIAS analyses coincide with other studies, showing a close relationship between crustaceans, mites, Ascaris, and cockroaches, with identity percentages ranging from 68% to 94% [4,5,13]. The elevated percentage of identity among tropomyosin sequences in these species is by amino acid sequence analysis, indicating the potential existence of IgE cross-reactive epitopes and the close taxonomic relationship between these species [4,8,11].

The B- and T-cell epitopes of tropomyosin from several species involved in allergenicity and cross-reactivity have been widely researched [5,15]. This study identified seven best candidates for B-cell epitopes and twenty-eight for linear T-cell epitopes. Several of these peptides overlapped with each other. The lineal B-07 peptide stands out for its significant conservation among HDM, crustaceans, cockroaches, and Ascaris species, with identity percentages varying from 91% to 100%. However, it is essential to note that peptide B-07, as well as peptides B01, B02a,b, and B04, have previously been identified by Ayuso and other researchers as significant regions of IgE binding and cross-reactivity epitopes among shrimp, cockroaches, HDM, Ascaris, and mosquitoes [5,11,40,41]. 

Interestingly, the linear B epitopes B05 (160RKYDEVARKLAMVEA174) and B06 (192ELEEELRVVGNNLKSLEVSEEKANQREEAYKE223) are new epitopes found in our analysis. Due to their high conservation among crustaceans, HDM, cockroaches, and Ascaris, these candidate peptides could be new potential epitopes participating in cross-reactivity. However, the accuracy of these epitopes requires confirmation in further experiments.

Remarkably, the number of conserved epitopes among the crustacean group (Lit v 1, Pen m 1, and Pen a 1) was significantly higher than among the mite, cockroach, and Ascaris tropomyosin and mosquito species. 

The investigation of cross-reactive T-cell epitopes has been limited to only a few allergens. On the other hand, T-cell cross-reactivity can be attributed to various factors, including the plasticity of MHC binding and the expansive recognition capacity of T-cell receptors (TCRs) [42]. It is known that T-cell epitopes with cross-reactivity can activate memory T cells and, subsequently, IgE production [43]. 

From this standpoint, we have employed bioinformatics tools to predict potential linear T epitopes and their MHC-binding HLA alleles that may contribute to cross-reactivity. However, it is imperative to note that the selection of these epitopes was based solely on their binding affinity to specific MHC molecules, employing a cutoff criterion of total scores (0.25) for epitope selection.

The most promising linear T-peptide candidates, exhibiting a high consensus among the ten species, are T15, T16, T17, T20, and T21. Remarkably, these peptides share overlapping amino acid sequences, differing by only one to two residues within these regions. These minor variations led to substantial shifts in conservation percentages among species. Intriguingly, these amino acid disparities appear to confer varying binding affinities for distinct HLA molecules. These discrepancies in consensus percentages predominantly stem from the presence or absence of specific amino acids, such as glutamic acid, leucine, arginine, and lysine [44,45]. In the context of proteins and peptides, it is widely recognized that the interaction of IgE antibodies with epitopes relies heavily on crucial amino acids that significantly influence the three-dimensional structure and stability of the antibody’s binding regions. Consequently, the precise arrangement of leucine (L), an uncharged hydrophobic amino acid, and the presence of glutamic acid (E), a negatively charged amino acid, assumes a critical role in epitope recognition by facilitating specific conformational interactions with other amino acids and maintaining secondary, as well as by increasing the binding affinity between the antibody and the allergenic molecule [45,46]. Moreover, this comprehensive knowledge of pivotal amino acids holds the potential to assess the risk of cross-reactive allergies and guide the development of mutant recombinant hypoallergenic proteins. 

The IEDB analysis revealed important linear T-cell epitopes conserved among arthropods, crustaceous, and helminths. Interestingly, T17 epitopes displayed exceptional conservation with a 100% identity across various species such as HDM, cockroaches, shrimp, and Ascaris, exhibit a distinct specificity towards the HLA-DQ alleles, particularly HLA-DQA1*03:01 and DQB103:02, suggesting that this particular sequence must have the ability to bind to diverse HLA types representing a broad cross-section of the population.

The IEDB’s Epitope Cluster Analysis of B- and T-cell epitope prediction showed an overlap between the T15 peptide and the B05 (RKYDEVARKLAMVE) (Consensus Core Epitope peptide) with high conservation between the species studied and specificity for the HLA allele DRB1*11:01 suggesting that this sequence could play a crucial role in the immune response orchestrated by both humoral and cell-mediated immunity (CD4+). A peptide with these properties could be useful in immunotherapy strategies. The ability to interact with both components of the immune system could be exploited to modulate immune responses and develop more specific therapeutic approaches.

Intriguingly, this peptide sequence has been previously documented as a T-cell epitope, rather than a B-cell epitope, within brown shrimp tropomyosin (Q3Y8M6), known to stimulate lymphoproliferation in individuals with a clinical history of allergies. A comprehensive assessment of the immunological activity of 18 peptides derived from brown shrimp tropomyosin was conducted by Ravkov E.V. et al., utilizing lymphoproliferation assays incorporating CFSE and tritiated thymidine. The outcomes of their study revealed that peptide RKYDEVARKLAMVE elicited a robust immune response and led to the release of proinflammatory cytokines, including IL-13 and IL-6 [47]. 

All candidate peptides derived from allergens were assessed using a comprehensive scoring system considering various criteria, including clinical relevance. This study implemented a pipeline to employ multiple tools for predicting T- and B-cell epitopes to identify consensus peptides across species with potential binding capacity to MHC class II molecules and IgE. However, this pipeline has limitations. Firstly, the tools used in this study to identify T- and B-cell epitopes rely primarily on TCR sequencing data or three-dimensional structure without considering experimentally validated epitopes. Secondly, the specific properties of amino acids and their position, crucial for the anchor region and affinity in defining peptides with immunogenic and antigenic properties in T lymphocytes, were not considered.

In summary, although our in silico analysis revealed pairs of candidate epitopes, including previously published and predicted peptides, the applied approach identified the consensus of new T and B epitopes between HDM, crustaceous, insects, and helminths, with their potential implication on the development of cross reactivity. The finding of these conserved target peptides and corresponding cross-reactive epitopes will help understand similar physio-pathological mechanisms in allergy diseases. In addition, our findings also demonstrate different patterns of conserved B-cell and T-cell binding epitopes among the ten tropomyosin species tested, suggesting that some TM-allergic patients will cross-react. Still, some peptides also showed species specificity, which could suggest species-specific biomarkers.

## 5. Conclusions

Allergenic cross-reactivity is crucial in triggering and developing allergic reactions. This study identified a consensus epitope between the T and B cells (B05 and TE-15) as the most promising cross-reactive peptide between mites, shrimp, cockroaches, and helminth species. Nonetheless, these observations are based on predictions generated through bioinformatics. Thus, it is imperative to evaluate their biological and immunological functionalities through a comprehensive approach involving in vitro experiments and in vivo investigations employing murine models in ex vivo and in vitro human cells. This novel in silico investigation aims to understand the phenomenon of cross-reactivity between these species, providing new and significant information that would significantly enhance our understanding of clinical cross-sensitization mechanisms and cross-reactivity. Additionally, this would facilitate the design of more effective diagnostic assays and treatments.

## Figures and Tables

**Table 1:** Tropomyosins from different sources included in this study.

Allergen	Organism	Uniprot N°	Allergome	Aa	kDa	3D Structure
Der p 10	*Dermatophagoides pteronyssinus*	O18416	Der p 10.0101	284	32.9	AlphaFold DB: https://alphafold.ebi.ac.uk/entry/O18416 accession on 21 July 2023
						Swiss-Model Repository: https://swissmodel.expasy.org/repository/uniprot/O18416?csm=278AAF663E585DB9 accession on 21 July 2023
Der f 10	*Dermatophagoides farinae*	Q23939	Der f 10.0101	284	32.9	AlphaFold DB: https://alphafold.ebi.ac.uk/entry/Q23939 accession on 21 July 2023
						Swiss-Model Repository: https://swissmodel.expasy.org/repository/uniprot/Q23939?csm=E665D29BAAF33507 accession on 21 July 2023
Blo t 10	*Blomia tropicalis*	A7XZI4 *	Blo t 10.0102	284	33	AlphaFold DB: https://alphafold.ebi.ac.uk/entry/A7XZI4 accession on 21 July 2023
						Swiss-Model Repository: https://swissmodel.expasy.org/repository/uniprot/A7XZI4?csm=C80E361A65A996F4 accession on 21 July 2023
Asc l 3	*Ascaris lumbricoides*	C0L3K2 *	Asc l 3.0101	287	33.5	AlphaFold DB: https://alphafold.ebi.ac.uk/entry/C0L3K2 accession on 21 July 2023
						Swiss-Model Repository: https://swissmodel.expasy.org/repository/uniprot/C0L3K2?csm=911B0C202DE1EB3F accession on 21 July 2023
Bla g 7	*Blattella germanica*	Q9NG56	Bla g 7.0101	284	32.8	AlphaFold DB: https://alphafold.ebi.ac.uk/entry/Q9NG56 accession on 21 July 2023
						Swiss-Model Repository: https://swissmodel.expasy.org/repository/uniprot/Q9NG56?csm=71DB260E3F594500 accession on 21 July 2023
Per a 7	*Periplaneta americana*	P0DSM6	Per a 7.0101	284	32.7	AlphaFold DB: https://alphafold.ebi.ac.uk/entry/P0DSM6 accession on 21 July 2023
						Swiss-Model Repository: https://swissmodel.expasy.org/repository/uniprot/P0DSM6?csm=1B91486A94446CF1 accession on 21 July 2023
Aed a 10	*Aedes aegypti*	Q17H82 *	Aed a 10.0101	284	32.3	AlphaFold DB: https://alphafold.ebi.ac.uk/entry/Q17H82 accession on 21 July 2023
Lit v 1	*Litopenaeus vannamei*	B4YAH6 *	Lit v 1.0101	284	32.9	AlphaFold DB: https://alphafold.ebi.ac.uk/entry/B4YAH6 accession on 21 July 2023
						Swiss-Model Repository: https://swissmodel.expasy.org/repository/uniprot/B4YAH6?csm=BE53B602C37E85E2 accession on 21 July 2023
Pen m 1	*Penaeus monodon*	A1KYZ2	Pen m 1.0101	284	32.8	AlphaFold DB: https://alphafold.ebi.ac.uk/entry/A1KYZ2 accession on 21 July 2023
						Swiss-Model Repository: https://swissmodel.expasy.org/repository/uniprot/A1KYZ2?csm=BE53B602C37E85E2 accession on 21 July 2023
Pen a 1	*Penaeus aztecus*	Q3Y8M6	Pen a 1.0102	284	32.8	AlphaFold DB: https://alphafold.ebi.ac.uk/entry/Q3Y8M6 accession on 21 July 2023
						Swiss-Model Repository: https://swissmodel.expasy.org/repository/uniprot/Q3Y8M6?csm=BE53B602C37E85E2 accession on 21 July 2023

*: UniProtKB unreviewed (TrEMBL) tropomyosin from different sources included in this study. Accession numbers (Allergome, UniProt): *Dermatophagoides pteronyssinus* (Der p 10), O18416; *Dermatophagoides farine* (Der f 10), Q23939; *Blomia tropicalis* (Blo t 10), A7XZI4; *Ascaris lumbricoides* (Asc l 3), C0L3K2; *Blattella germanica* (Bla g 7), Q9NG56; *Periplaneta americana* (Per a 7), P0DSM6; *Aedes aegypti* (Aed a 10), Q17H82; *Litopenaeus vannamei* (Lit v 1), B4YAH6; *Penaeus monodon* (Pen m 1), A1KYZ2; and *Penaeus aztecus* (Pen a 1), Q3Y8M6.

**Table 2:** B-cell epitopes (IgE and lineal epitopes) are highly conserved. The final list of linear B-cell epitopes predicted for ten allergens used the AlgPred 2.0 and BepiPred 3.0 server. The epitope allergen sources, their amino acid sequences, and positions in the protein by cluster analysis are presented here. The degree of conservation of the epitopes within the sequences of the respective allergens was calculated using the conservancy analysis tool on the IEDB website.

	Residue				Identity %									
B-Epitope ID	Start	End	Length	Consensus Sequences	Asc l 3	Aed a 10	Der p 10	Der f 10	Blo t 10	Lit v 1	Pen m 1	Pen a 1	Per a 7	Bla g 7
BE-01	48	55	8	KRMQQLEN	63	63	63	63	63	100	100	100	63	63
BE-02a	85	104	20	VAALNRRIQLLEEDLERSEE	80	70	95	95	95	100	100	100	100	100
BE-02b	85	104	20	VAALNRRIQLIEEDLERSEE	75	70	100	100	100	95	95	95	95	95
BE-03	128	141	14	KVLENRSQQDEERM	79	100	71	71	71	86	86	86	57	57
BE-04	132	153	22	NRSLSDEERMDALENQLKEARF	59	77	73	77	73	100	100	100	82	82
BE-05	160	174	15	RKYDEVARKLAMVEA	100	67	100	100	100	100	100	100	93	93
BE-06	192	223	32	ELEEELRVVGNNLKSLEVSEEKANQREEAYKE	88	78	88	91	91	100	100	100	91	91
BE-07	251	261	11	KEVDRLEDELV	100	91	91	100	100	100	100	100	100	100

**Table 3:** T-cell epitopes conserved with strong binding (<0.5) to MHC-II. Final T-cell epitopes predicted used the IEDB MHC-II binding prediction tool. The epitope sequences, amino acid position and length, and T-cell epitopes’ corresponding HLA-binding alleles are presented here. The degree of conservation of the epitopes within the sequences of the respective allergens was calculated using the conservancy analysis tool on the IEDB website.

					Identity %									
T-Epitope ID	MHC-II Alleles	Consensus Sequence	Start	End	Asc l 3	Aed a 10	Der p 10	Der f 10	Blo t 10	Lit v 1	Pen m 1	Pen a 1	Bla g 7	Per a 7
TE-01	HLA-DRB1*04:01 HLA-DRB1*04:04	KMQAMKLEKDNAADKAD--	7	23	82	100	82	82	82	88	88	88	82	82
TE-02	HLA-DRB1*03:01	-MQAMKIEKDNALDRADAA	8	25	100	72	78	78	78	78	78	78	72	67
TE-03	HLA-DRB1*03:01	--QAMKLEKDNAIDRAEI-	9	24	75	75	100	100	100	81	81	81	81	75
TE-04	HLA-DRB1*03:01	--QAMKLEKDNAMDRADT-	9	24	81	88	81	81	81	100	100	100	88	81
TE-05	HLA-DQA1*03:01/DQB1*03:02 HLA-DQA1*05:01/DQB1*02:01	ARDANIRAEKAEEEARS	29	45	41	47	76	76	71	53	53	53	100	94
TE-06	HLA-DRB1*11:01 HLA-DRB1*11:04 HLA-DRB1*04:04 HLA-DRB1*04:05	KSEEEVRALQKKIQQIENELDQVQE	38	62	56	40	100	100	100	76	76	76	76	76
TE-07	HLA-DRB1*04:05	-------SLQKKIQQIENDLDQTM-	45	61	53	41	76	76	76	65	65	65	100	100
TE-08	HLA-DRB1*11:04	SEEEVHNLQKRMQQLE	39	54	50	44	69	69	69	100	100	100	56	56
TE-09	HLA-DRB1*04:05	AQEDLSVANSNLEEKE	60	75	100	50	69	69	63	50	50	50	38	38
TE-10	HLA-DRB1*14:01 HLA-DRB1*11:04	LKANIQLVEKDKALSNAE	65	82	44	44	56	56	50	100	100	100	61	61
TE-11	HLA-DRB1*11:01 HLA-DRB1*11:04	STEANVAALTRKVQQVEE	80	97	50	100	50	50	50	50	50	50	50	50
TE-12	HLA-DRB1*11:04 HLA-DRB1*11:01	TAEGDVAALNRRIQLIE	80	96	65	47	100	100	100	82	82	82	76	76
TE-13	HLA-DRB1*11:04	NAESEVAALNRRIQLLE	80	96	76	47	76	76	76	94	94	94	100	100
TE-14	HLA-DRB1*11:04 HLA-DRB1*11:01	NAEGEVAALNRRIQLLE	80	96	76	47	82	82	82	100	100	100	94	94
TE-15	HLA-DRB1*11:01	ADRKYDEVARKLAMVE	158	173	100	75	100	100	100	100	100	100	94	94
TE-16	HLA-DRB1*11:01	ADKKYDEVARKLAMVE	158	173	94	75	94	94	94	94	94	94	100	100
TE-17	HLA-DQA1*03:01/DQB1*03:02	VARKLAMVEADLERAE	165	180	100	69	100	100	100	100	100	100	100	100
TE-18	HLA-DRB1*04:05 HLA-DQA1*03:01/DQB1*03:02 HLA-DRB1*03:01	VGNSLKSLEVSEDKANQRVEE	200	220	71	100	76	76	76	81	81	81	81	81
TE-19	HLA-DQA1*03:01/DQB1*03:02	VGNNLKSLEVSEEKAL-----	200	215	100	81	94	94	94	94	94	94	94	94
TE-20	HLA-DQA1*03:01/DQB1*03:02 HLA-DRB1*04:05	VGNNLKSLEVSEEKAQ-----	200	215	94	81	100	100	100	94	94	94	94	94
TE-21	HLA-DQA1*03:01/DQB1*03:02 HLA-DRB1*04:05	VGNNLKSLEVSEEKAN-----	200	215	94	88	94	94	94	100	100	100	100	100
TE-22	HLA-DRB1*11:01	REDSYEEQIRTVSAR	217	231	100	27	40	53	47	47	47	47	47	47
TE-23	HLA-DRB1*11:04	ERSVQKLQKEVGRLE	243	257	93	73	100	93	87	93	93	93	93	93
TE-24	HLA-DQA1*05:01/DQB1*02:01 HLA-DRB1*04:01 HLA-DRB1*04:05 HLA-DQA1*03:01/DQB1*03:02	VHEKEKYKSISDELDQTFA	261	279	84	53	100	100	100	84	84	84	74	74
TE-25	HLA-DQA1*05:01/DQB1*02:01 HLA-DRB1*04:01 HLA-DRB1*04:05 HLA-DQA1*03:01/DQB1*03:02	VNEKEKYKSITDELDQTFS	261	279	74	47	84	84	84	100	100	100	68	68
TE-26	HLA-DRB1*04:05 HLA-DQA1*03:01/DQB1*03:02 HLA-DQA1*05:01/DQB1*02:01	-HEKERYKSISEELDQTF-	262	278	100	53	88	88	88	76	76	76	65	65
TE-27	HLA-DRB1*04:05	--EKEKYKYICDDLDMTF-	263	278	63	44	75	75	75	75	75	75	100	94
TE-28	HLA-DRB1*04:05 HLA-DQA1*03:01/DQB1*03:02	VNKDRYKSLADEMDSTFA	261	279	50	100	56	56	56	50	50	50	39	39

**Table 4:** Conservation analysis of final B-cell and T-cell consensus epitope sequences. Final B-cell and T-cell consensus epitopes were used in the IEDB’s Epitope Cluster Analysis tool.

Cluster Number	Epitope Number	B-Cell and T-Cell Consensus Epitope Sequence	Peptide
1.1	Consensus	ELEEELRVVGNNLKSLEVSEEKANQRXEXYKE	-
1.1	1	ELEEELRVVGNNLKSLEVSEEKANQREEAYKE	B06
1.1	2	--------VGNSLKSLEVSEDKANQRVEE---	TE-18
1.1	3	--------VGNNLKSLEVSEEKAL--------	TE-19
1.1	4	--------VGNNLKSLEVSEEKAQ--------	TE-20
1.1	5	--------VGNNLKSLEVSEEKAN--------	TE-21
2.1	Consensus	NAEGEVAALNRRIQLLEEDLERSEE	-
2.1	1	TAEGDVAALNRRIQLIE--------	TE-12
2.1	2	NAESEVAALNRRIQLLE--------	TE-13
2.1	3	NAEGEVAALNRRIQLLE--------	TE-14
2.1	4	-----VAALNRRIQLLEEDLERSEE	B02a
2.1	5	-----VAALNRRIQLIEEDLERSEE	B02b
3.1	Consensus	KMQAMKLEKDNAXDRADXA	-
3.1	1	KMQAMKLEKDNAADKAD--	TE-01
3.1	2	-MQAMKIEKDNALDRADAA	TE-02
3.1	3	--QAMKLEKDNAIDRAEI-	TE-03
3.1	4	--QAMKLEKDNAMDRADT-	TE-04
4.1	Consensus	VHEKEKYKSIXDELDQTFX	-
4.1	1	VNEKEKYKSITDELDQTFS	TE-25
4.1	2	VHEKEKYKSISDELDQTFA	TE-24
4.1	3	-HEKERYKSISEELDQTF-	TE-26
4.1	4	--EKEKYKYICDDLDMTF-	TE-27
5.1	Consensus	ADRKYDEVARKLAMVEA	-
5.1	1	ADRKYDEVARKLAMVE-	TE-15
5.1	2	ADKKYDEVARKLAMVE-	TE-16
5.1	3	--RKYDEVARKLAMVEA	B05
6.1	Consensus	SEEEVHNLQKRMQQLEN	-
6.1	1	SEEEVHNLQKRMQQLE-	TE-08
6.1	2	---------KRMQQLEN	B01
7.1	Consensus	KSEEEVRXLQKKIQQIENXLDQXXE	-
7.1	1	KSEEEVRALQKKIQQIENELDQVQE	TE-06
7.1	2	-------SLQKKIQQIENDLDQTM-	TE-07
8.1	Singleton	KVLENRSQQDEERM	B03
9.1	Singleton	NRSLSDEERMDALENQLKEARF	B04
10.1	Singleton	STEANVAALTRKVQQVEE	TE-11
11.1	Singleton	ERSVQKLQKEVGRLE	TE-23
12.1	Singleton	VNKDRYKSLADEMDSTFA	TE-28
13.1	Singleton	KEVDRLEDELV	B07
14.1	Singleton	ARDANIRAEKAEEEARS	TE-05
15.1	Singleton	AQEDLSVANSNLEEKE	TE-09
16.1	Singleton	LKANIQLVEKDKALSNAE	TE-10
17.1	Singleton	REDSYEEQIRTVSAR	TE-22
18.1	Singleton	VARKLAMVEADLERAE	TE-17

## Data Availability

Data are contained within the article and supplementary materials.

## Supplementary Materials

The following supporting information can be downloaded at https://www.mdpi.com/article/10.3390/biomedicines12040884/s1. Supplementary Materials: prediction.
